# Monitoring of PM_2.5_ Concentrations by Learning from Multi-Weather Sensors

**DOI:** 10.3390/s20216086

**Published:** 2020-10-26

**Authors:** Yuexia Wang, Zhihuo Xu

**Affiliations:** Radar Remote Sensing Group, School of Transportation, Nantong University, Nantong 226019, China; venus@ntu.edu.cn

**Keywords:** particulate matter, meteorological parameters, multivariate linear regression, multivariate nonlinear regression, neural network, machine learning

## Abstract

This paper aims to monitor the ambient level of particulate matter less than 2.5 μm (PM${}_{2.5}$) by learning from multi-weather sensors. Over the past decade, China has established a high-density network of automatic weather stations. In contrast, the number of PM monitors is much smaller than the number of weather stations. Since the haze process is closely related to the variation of meteorological parameters, it is possible and promising to calculate the concentration of PM${}_{2.5}$ by studying the data from weather sensors. Here, we use three machine learning methods, namely multivariate linear regression, multivariate nonlinear regression, and neural network, in order to monitor PM${}_{2.5}$ by exploring the data of multi-weather sensors. The results show that the multivariate linear regression method has the root mean square error (RMSE) of 24.6756 μg/m${}^{3}$ with a correlation coefficient of 0.6281, by referring to the ground truth of PM${}_{2.5}$ time series data; and the multivariate nonlinear regression method has the RMSE of 24.9191 μg/m${}^{3}$ with a correlation coefficient of 0.6184, while the neural network based method has the best performance, of which the RMSE of PM${}_{2.5}$ estimates is 15.6391 μg/m${}^{3}$ with the correlation coefficient of 0.8701.

## 1. Introduction

Particulate matter (PM) is a kind of atmospheric aerosol, formatting as minute solid particles or liquid droplets suspended in air [1]. PM is mainly from anthropogenic origin, derived from industrial, home heating and cooking, and transportation sources while most natural sources are relatively less important [2]. PM less than 10 μm (PM${}_{10}$) and PM less than 2.5 μm (PM${}_{2.5}$) consist of a number of components such as sulfate, nitrate, ammonium, elemental carbon, organic carbon, and soil or dust particles. Fine particles, PM${}_{1.0}$ (aerodynamic diameter of less than 1.0 μm), carry toxic trace elements, like Se, S, V, Cu, Fe, Pb, As, Cd, Ni, Zn, Mn, etc. [3]. Scientific studies reveal that these PM increase the risk of anthroposphere, atmosphere, hydrosphere, biosphere, and lithosphere [2,3,4]. The negative impact of PM can be summarized as follows: first, it affects human health [5,6,7]. PM${}_{2.5}$ and PM${}_{10}$ generally passes through the nose and throat and even enters the lungs; fine particles, PM${}_{1.0}$, and smaller particles are able to penetrate into the human respiratory and circulation system, resulting in adverse health effects [4]. Second, PM episodes reduce visibility and lead to climate change [8]. PM is the main cause of reduced visibility (haze) in the world. It suppresses convection and precipitation by both radiative and micro-physical effects, changes lighting phenomenon in different regions, weakens the hydrological cycle, and leads to less fresh water and nutrient imbalance in coastal waters and large river basins [4]. Third, particle pollution and acid rain make lakes and streams acidic, damage sensitive forests, farm crops, stone, soil buildings and other materials, and deplete the nutrients in soil, affecting the diversity of ecosystems [9].

As particles pollution is becoming an increasingly severe problem, measurement and prediction of PM are crucial for environment protection and climate investigations. Since the 1970s, global aerosol monitoring programs using remote sensing tools have been launched to measure the level of component aerosol on the regional or global scale [10,11,12,13,14,15]. Launched on 13 October 2017, the European Space Agency (ESA) Sentinel-5 Precursor is a low-Earth orbit polar satellite designed to provide information and services on air quality, climate, and the ozone layer, as part of the Global Monitoring for Environment and Security Space Sub-Programme. The mission’s payload is the tropospheric monitor, which measures key atmospheric constituents, including ozone, nitrogen dioxide, sulphur dioxide, carbon monoxide, methane, nitrous oxide, and Ultraviolet aerosol index [16]. At the same time, ground based direct samplers were developed to accurately measure PM concentrations [17,18]. Zhang et al. [19] demonstrated how vertical wind shear affects ground-level PM${}_{2.5}$ by using radar wind profiler observations in Beijing, providing new insights into the role of vertical wind shear in modulating the variation of PM${}_{2.5}$, which is worth considering in future air quality predictions. In addition, airborne observations were conducted to advance the remote sensing of aerosols. Knobelspiesse et al. [20] conducted one field campaign that used the airborne hyper angular rainbow polarimeter, the airborne multiangle spectro-polarimetric imager, the airborne spectrometer for planetary exploration, and the research scanning polarimeter to test new observation systems, develop new algorithms, and validate orbital observations. Wang et al. [21] developed an unmanned aerial vehicle PM monitoring system that can performed three-dimensional stereoscopic observation of PM${}_{2.5}$ and PM${}_{10}$ in the atmosphere. These measurements significantly improve our understanding of the characteristics of PM.

Regarding predictions of PM concentrations, some methods have been developed. For example, a Bayesian based regression model by Mølgaard et al. [22], a Gaussian process regression method by Reggente et al. [23], and a multiple linear regression model have been applied for forecasting the fine particle concentrations based on measurements of nitrogen oxides. Using the raw data of air temperature, relative humidity and PM, Wang et al. [21] recently applied a multiple linear regression model, support vector machine, and random forest for correction of PM ${}_{2.5}$ and obtained satisfactory results. To reduce bias and improve non-reference monitoring data used for community air monitoring studies, Commodore et al. [24] proposed one nonlinear statistical model as an example of instrument evaluation prior to assessment of non-reference monitoring measures of PM. Based on satellite observations and ground monitor data, a geographically weighted regression model was applied to estimate global PM${}_{2.5}$ [25]. Donkelaar et al. [26] developed one geoscience-derived approach to estimate of PM${}_{2.5}$ composition over North America from 2000 to 2016. Hammer et al. estimated global PM${}_{2.5}$ concentrations and trends in the period of 1998–2018 by using satellite observations, chemical transport models, and ground-based monitoring [27].

In fact, the haze pollution process is closely related to the evolution of meteorological parameters [28]. First, PM concentrations are the main cause of reduced visibility with light extinction effects, which causes the haze phenomenon to arise. Visibility is a measure of the clearness of the atmosphere [29]. Regional haze reduces visibility by the presence of particles suspended in the atmosphere and is usually expressed in terms of light extinction or haze index. PM scatters and absorbs light substantially. It has been shown that particles of different size and chemical composition can affect visibility very differently. The fine sulfate and nitrate particles are the main contributors to haze, especially in the presence of water vapor. The contributions of elemental and organic carbon are also important in visibility degradation while most other particles are relatively less important. During PM episodes, the atmospheric visibility is less than 10 km due to the presence of PM in the atmosphere. Second, wind speed and wind direction of automatic weather stations (AWS) are generally used to assess how well mass transport is being characterized [30]. In general, the probability of PM episodes is low with high speed wind. Previous work has demonstrated that, if relative humidity (RH) is higher than 80%, the probability of PM episodes is also low. It is inferred that the high temperature will make the water of surface evaporate up into the air, which indirectly affects the PM cycle. In addition, the visibility also decreases in the presence of rain. Thus, it is necessary to supplement the visibility observation using precipitation measurements, in order to decide whether PM episodes really happen depending on the visibility measurements.

As addressed above, since the meteorological parameters can be affected by PM episodes, it is possible and promising to measure the concentration of PM by studying the data from weather sensors, even though AWS were not originally designed for PM observation. There is a high density distribution network of AWS in China, and these AWSs work 24/7 under all weather conditions. Because of the absence of dense urban PM monitoring networks, values observed at a ’central monitor’ were frequently considered to be representative for ambient pollutant levels within a metropolitan area. With recent growth of the high density network of AWS, we have an opportunity to measure PM more accurately based on data from AWS. However, little work has been done to calculate PM concentration by using meteorological parameters. Our previous study using hidden Markov models to quantify PM concentrations have yielded some encouraging results [31]. In this paper, we aim to use three machine learning methods, namely multivariate linear regression, multivariate nonlinear regression and neural network, to retrieve PM concentrations by learning from the data of multi-weather sensors.

## 2. Materials and Methods

### 2.1. Materials

**Figure 1:**
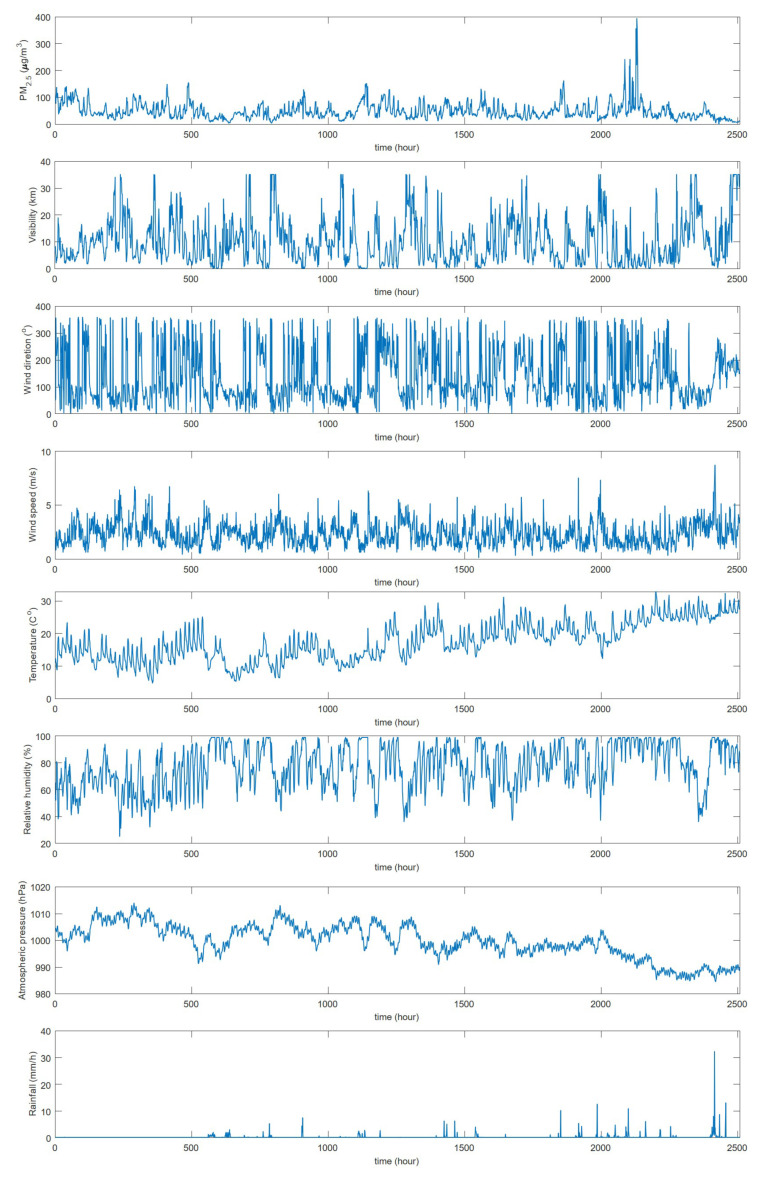
This figure shows temporal variations of PM${}_{2.5}$ concentrations and meteorological parameters at the National Xiamen weather station during the period of January 2014–June 2014.

**Figure 2:**
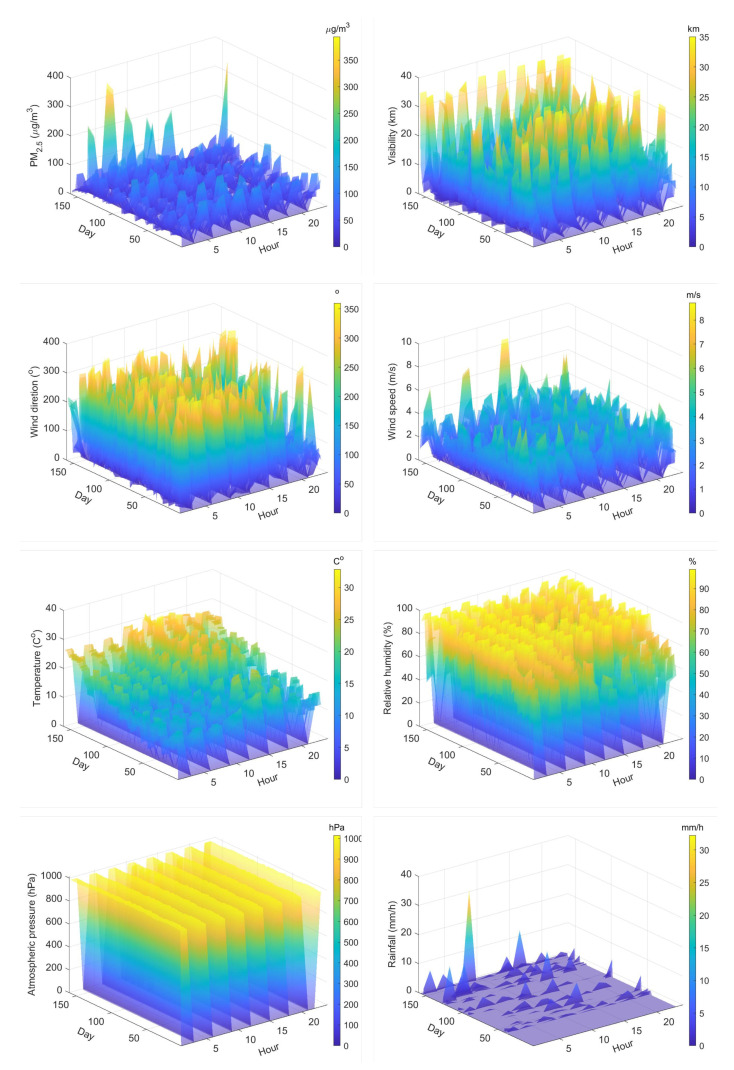
This figure shows two-dimensional temporal variations of PM${}_{2.5}$ concentrations and meteorological parameters by 24 h per day in the period of January 2014–June 2014.

Observations of PM${}_{2.5}$ and meteorological parameters were collected from January 2014 to June 2014 at the National Xiamen weather station, Fujian, China. The PM monitoring station was installed in the same standard observation site as the automatic multi-weather sensors. The meteorological parameters and PM${}_{2.5}$ data were collected at a frequency of once per hour and processed by using the world meteorological data quality control standard. The main meteorological parameters are visibility, wind direction, wind speed, temperature, relative humidity, atmospheric pressure, and hourly rainfall rate. Figure 1 shows variations of PM${}_{2.5}$ concentrations and meteorological parameters at the National Xiamen weather station during the coordinated observation period. For better visualization, we divided the PM${}_{2.5}$ data and meteorological parameters into two dimensions according to 24 h per day, as shown in Figure 2.

In order to study the relationship between PM${}_{2.5}$ and meteorological parameters, linear regressions were performed by using Pearson’s linear correlation. Pearson’s correlation coefficient is widely used to measure the degree of linear correlation between two quantitative variables [32]. Given *N* samples of two variables *x* and *y*, the coefficient $r_{xy}$ is calculated as

(1) $$r_{xy}=\frac{N\sum_{i=1}^{N}\left(x_{i}y_{i}\right)-\sum_{i=1}^{N}x_{i}\sum_{i=1}^{N}y_{i}}{\sqrt{N\sum_{i=1}^{N}x_{i}^{2}-{\left(\sum_{i=1}^{N}x_{i}\right)}^{2}}\sqrt{N\sum_{i=1}^{N}y_{i}^{2}-{\left(\sum_{i=1}^{N}y_{i}\right)}^{2}}}$$

where $x_{i}$ and $y_{i}$ are the *i*th sample points.

The results were reported in Table 1. PM${}_{2.5}$ has a high correlation with visibility, wind direction, wind speed, and relative humidity, and a low correlation with air temperature, atmospheric pressure, and rainfall rate. Therefore, we use the meteorological parameters with high correlation coefficients, namely visibility, wind direction, wind speed and relative humidity, in order to develop the multivariate regression model.

Interestingly, we also found that the performance of neural network based method can be improved by using these meteorological parameters, even though relative humidity, atmospheric pressure, and rainfall rate have low correlation with PM${}_{2.5}$. Thus, all seven meteorological parameters were used in the neural network method.

### 2.2. Machine Learning Methods

#### 2.2.1. Multivariate Linear Regression

Denote the *i*th observation of PM${}_{2.5}$, visibility, wind direction, wind speed, and relative humidity as *y${}_{i}$*, *X${}_{i}^{1}$*, *X${}_{i}^{2}$*, *X${}_{i}^{3}$*, and *X${}_{i}^{4}$*, respectively, we predict the PM${}_{2.5}$ via the model as

(2) $$\hat{y}=a_{0}+\sum_{k=1}^{4}X_{i}^{k}a_{k}$$

where $a_{0}$ is the intercept. We include $a_{0}$ in the vector of coefficients ***a***, Equation (1) can be written in vector form as an inner product

(3) $$\hat{y}=X^{T}a$$

The optimal vector of coefficients $\hat{a}$ can be generated by minimizing the distance between the predictions and the ground truth data. Using Euclidean distance, the solution of the vector of coefficients can be formulated as

(4) $$\hat{a}=\underset{a}{\arg\min}∥y-X^{T}{a∥}_{2}^{2}$$

where **y** is a vector of PM${}_{2.5}$ data in the training set.

The least squares method was applied to fit the above model, and the solution is given by using the Moore–Penrose inverse operation as

(5) $$\hat{a}={\left(X^{T}X\right)}^{-1}X^{T}y$$

#### 2.2.2. Multivariate Nonlinear Regression

**Figure 3:**
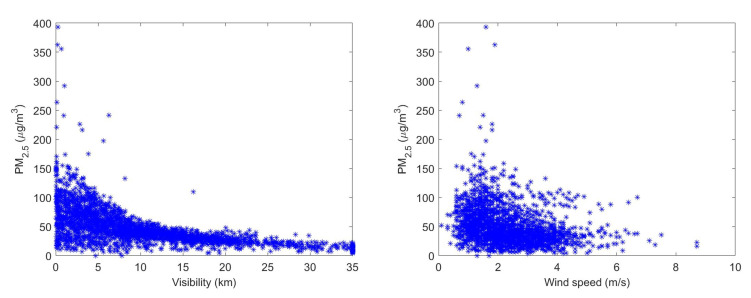
This figure shows nonlinear distribution of PM${}_{2.5}$ concentrations with visibility and wind speed, respectively.

The physical principle behind our proposed nonlinear regression model is that the value of atmospheric optical visibility decays as an exponential function with increasing of PM concentrations. In addition, as the wind speed increases, it becomes easier for the particles matter to disperse and the concentration becomes smaller. Figure 3 shows this physical nonlinear relationship. The nonlinear regression model is given by

(6) $$\hat{y}=\sum_{k=1}^{4}\gamma_{k}\exp\left(-\beta_{k}X_{i}^{k}\right)$$

where $\gamma_{k}$ and $\beta_{k}$ are the *k*th coefficients of the model.

In this approach, we pick the coefficientsγ and β to minimize the cost function as residual sum of squares as

(7) $$f\left(\theta \right)=\sum_{i=1}^{N}{\left(y_{i}-\sum_{k=1}^{4}\gamma_{k}e^{\left(-\beta_{k}X_{i}^{k}\right)}\right)}^{2}$$

where *N* is total number of data samples, the parameters θ=(γ,β).

The above problem was solved by using the Nelder–Mead simplex method [33,34]. One vector of the parameters θ represents one simplex. The major procedures of the Nelder–Mead simplex method include order, reflection, expansion, contraction, and shrink operation. Algorithm 1 summarizes the detailed procedures for fitting the multivariate nonlinear regression model.
**Algorithm 1:** Nelder–Mead simplex method for multivariate nonlinear regression
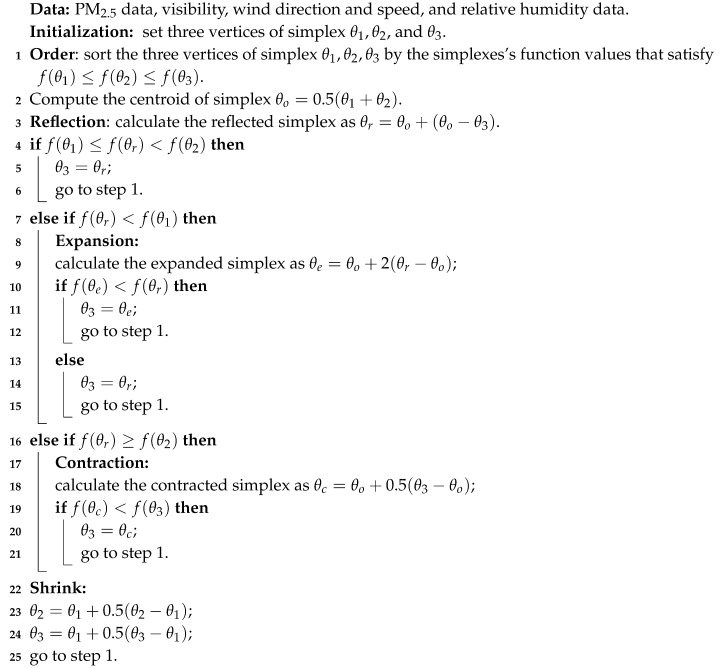


#### 2.2.3. Neural Network

**Figure 4:**
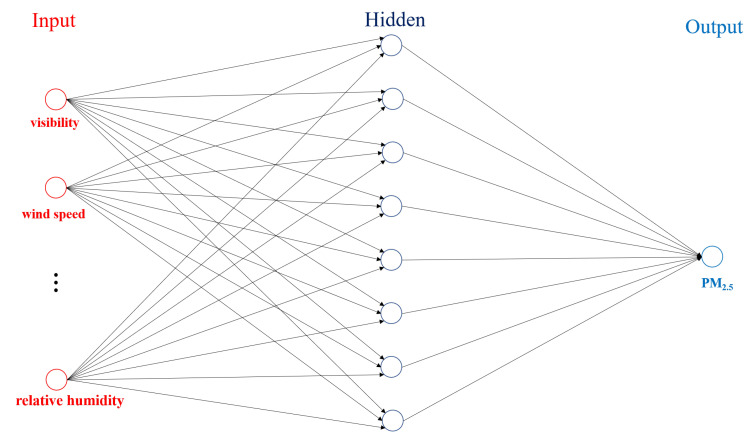
This figure shows a conceptualized diagram of a two-layer feed-forward network that is used for predicting PM concentrations.

Neural networks are particularly well suited to dealing with nonlinear fitting problems, due to the fact that enough elements (called neurons) can fit any data with arbitrary precision [35,36]. A multilayer perception (MLP) network [37] is applied to explore the nonlinear regression for PM${}_{2.5}$. Figure 4 shows a conceptualized structure of a two-layer feed-forward network that is used for predicting PM concentrations.

The proposed two-layer feed-forward neural network includes a sigmoid hidden layer and an affine transformation output layer. Assuming that the number of entries in the hidden layer is $m_{h}$, the input–output function can be formulated as

(8) $$f_{\theta }\left(X\right)=b+v\mathrm{sigmoid}\left(c+Wx\right)$$

where x is defined above, *b* is the output offset, v is the weight vector for the output layer, $\mathrm{sigmoid}\left(x\right)=1/\left(1+e^{-x}\right)$, c is the offset vector for the hidden layer, and W is the weight matrix for the hidden layer, and the parameters θ=(b,c,v,W). As mentioned above, all seven meteorological parameters were input in the neural network. Thus, the dimension of the parameters can be determined as $c\in R^{m_{h}}$, $v\in R^{m_{h}}$, $W\in R^{m_{h}\times 7}$.

To avoid over-fitting the data, we applied the Bayesian regularization method to train the network [38,39]. Firstly, the sum of squares of the vector weights is defined as

(9) $$E_{W}=\alpha {\left(\sum_{i=1}^{m_{h}}\sum_{k=1}^{7}W_{ik}^{2}+\sum_{i=1}^{m_{h}}v_{i}\right)}^{2}$$

where α is the parameters of the function.

In addition, the sum of squared errors is given

(10) $$E_{D}=\beta \sum {\left(y-(b+v\mathrm{sigmoid}(c+Wx))\right)}^{2}$$

where β is the parameters of the error function.

From the perspective of Bayesian framework, the weights of neural network are considered as stochastic variables. According to Bayes’ rule, the probability density function of the parameters for a neural network *M* can be formulated as

(11) $$p\left(\theta |D,\alpha ,\beta ,M\right)=\frac{p(D|\theta ,\alpha ,\beta ,M)p(\theta |\alpha ,\beta ,M)}{p(D|\alpha ,\beta ,M)}$$

Specifically, the likelihood function does not depend on the regularizer α once the parameters θ is known, and the prior function does not depend on the parameter β that regularizes the data term [38]. Therefore, the above equation can be simplified as

(12) $$p\left(\theta |D,\alpha ,\beta ,M\right)=\frac{p(D|\theta ,\beta ,M)p(\theta |\alpha ,M)}{p(D|\alpha ,\beta ,M)}$$

Assuming that the prior of the parameters θ and the training data are Gaussian distributed, the probability density functions can be represented

(13) $$p\left(D|\theta ,\beta ,M\right)=\frac{\exp(-\beta E_{D})}{{\left(\pi /\beta \right)}^{N_{D}/2}}$$

where $N_{D}$ is the total number of training data samples.

In addition,

(14) $$p\left(\theta |\alpha ,M\right)=\frac{\exp(-\alpha E_{W})}{{\left(\pi /\alpha \right)}^{N_{W}/2}}$$

where $N_{W}$ is the number of the weights.

The optimal parameters of the neural network can be obtained by maximizing the posterior probability (Equation (12)). Substituting Equations (13) and (14) into Equation (12), we can obtain

(15) $$p\left(\theta |D,\alpha ,\beta ,M\right)\propto \frac{1}{Z(\alpha ,\beta )}\exp\left(-\alpha E_{W}-\beta E_{D}\right)$$

where $\frac{1}{Z(\alpha ,\beta )}$ is the normalization factor. According to the above derivation, maximizing the above posterior probability is equivalent to minimized the regularized cost function as

(16) $$J\left(\theta \right)=\alpha E_{W}+\beta E_{D}$$

Here, we use one approach of Gauss–Newton approximation for Bayesian regularization [39]. The more details of Bayesian regularization for neural network can also be found in [40,41,42,43].

## 3. Results

### 3.1. Models Training

After solving Equation (4) by least squares, the multivariate linear regression model was determined to be

(17) $$\begin{array}{cc} {\mathrm{PM}}_{2.5}= & 113.93-0.0024\times Visibility+0.051\times \mathrm{Wind}\mathrm{direction}\\ \; & -2.962\times \mathrm{Wind}\mathrm{speed}-0.5336\times RH \end{array}$$

**Figure 5:**
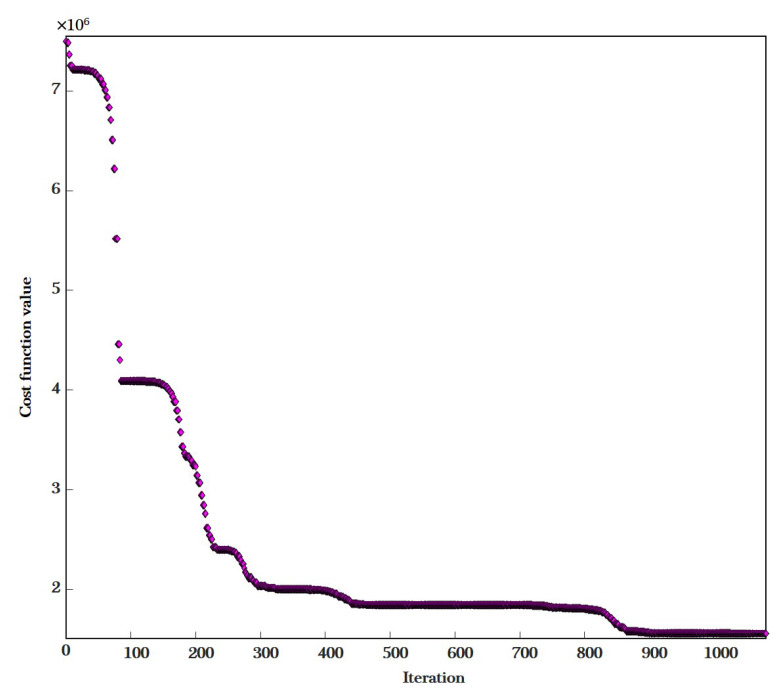
This figure shows variations of the cost function value during the training of the multiple nonlinear regression model, using Algorithm 1. The value of the cost function is calculated by using Equation (7).

Next, we describe the training process of the multivariate nonlinear regression model. As shown in Figure 5, the cost function value decreased rapidly during the first 300 iterations by using Algorithm 1. When the number of iterations reached 900, the performance of the algorithm approached saturation. Finally, the multivariate nonlinear regression model was completely fitted after 1081 iterations as

(18) $$\begin{array}{cc} {\mathrm{PM}}_{2.5}= & 68.7672e^{\left(-0.0001\times Visibility\right)}+8.3784e^{\left(0.0029\times \mathrm{Wind}\mathrm{direction}\right)}\\ \; & -0.0536e^{\left(0.7548\times \mathrm{Wind}\mathrm{speed}\right)}+7.3861e^{\left(-0.256\times RH\right)} \end{array}$$

In the training of neural network models, validation is not required due to the use of the Bayesian regularization method. Therefore, the PM data and meteorological parameters were randomly divided into two sets as: 60% for training, and 40% for a completely independent test. The number of these datasets is 70% of all data, meaning that 42% of the data has been used for training. Validation is often considered as a form of regularization to meet the balance between under-fitting and over-fitting. Interestingly, the Bayesian regularization method has its own form of validation built into the approach [38,39], so this paradigm disables validation of the dataset, since the purpose of checking validation is to see if the error on the validation set gets better or worse as training progresses. The error of Bayesian regularization is based not only on how the model behaves on the dataset, but also on the size of the weights in the hidden layers. The larger the weights, the larger the error. Thus, throughout the training process, the hidden layer may never be allowed to explore larger weights, even if larger weights may result in a global minimum.

**Figure 6:**
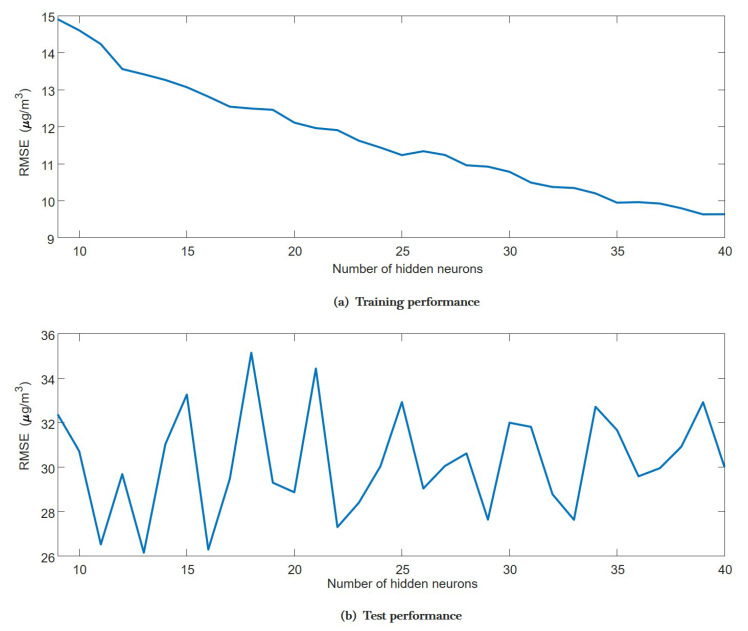
This figure shows variations of the training performance (**a**) and the test performance (**b**) with respect to the number of hidden neurons.

We averaged the training and test performance over 100 experiments on models with the different numbers of hidden neurons, and the results are shown in Figure 6. These results support the issues mentioned above. While increasing the number of hidden neurons can improve the performance of training, it does not reduce the error for testing. Therefore, by considering the training and test performance together, we set the number of hidden neurons as 16, which can produce satisfactory performance.

### 3.2. Predictions of PM${}_{2.5}$ Concentrations

**Figure 7:**
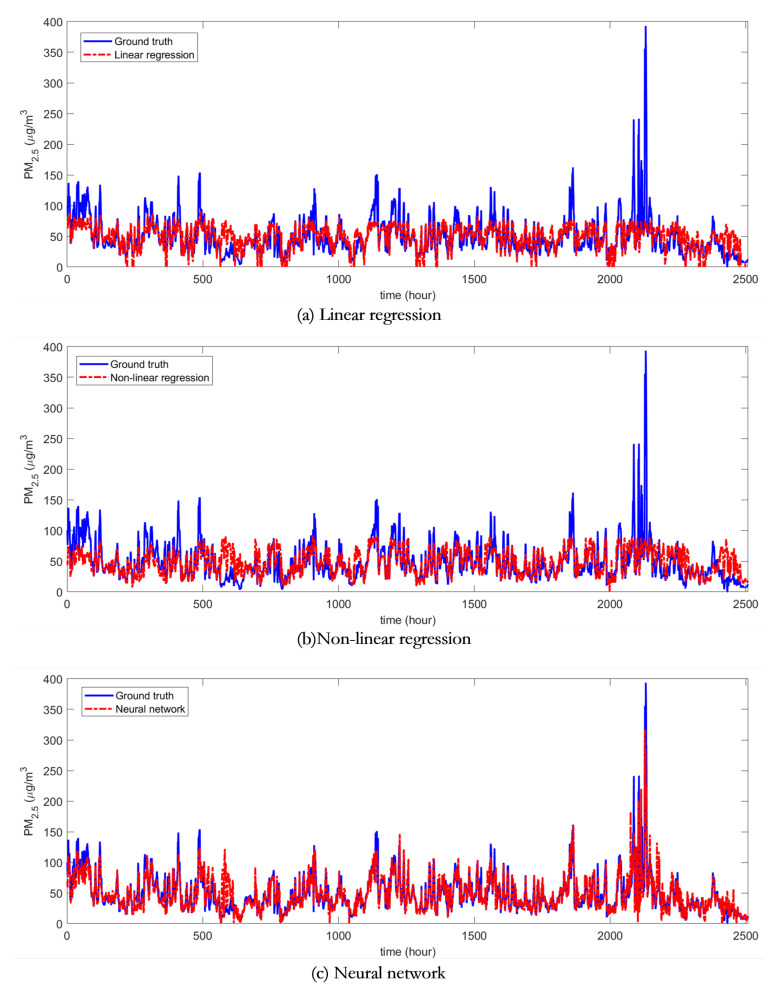
This figure shows the PM${}_{2.5}$ concentrations estimated by using the different machine learning methods.

**Figure 8:**
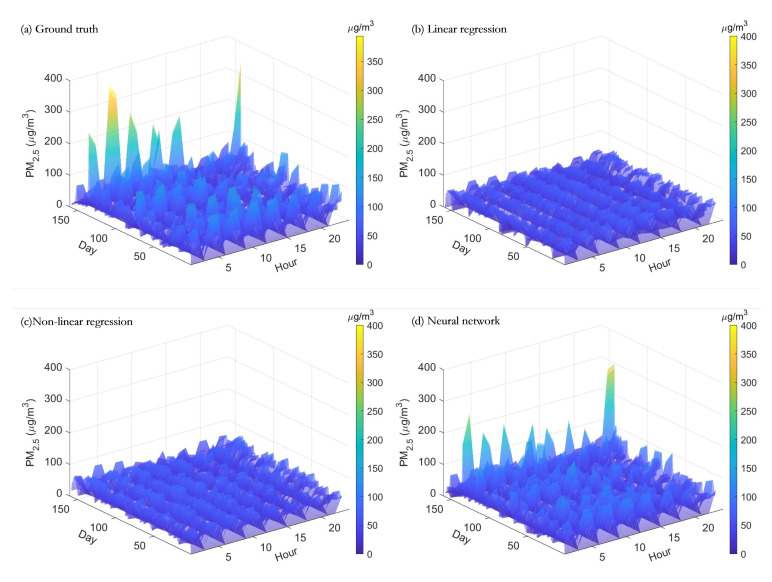
This figure shows comparisons of PM${}_{2.5}$ concentrations estimated using different machine learning methods.

Using these three trained models, the meteorological parameters were input to obtain the estimated PM concentrations, which were shown in Figure 7 and Figure 8, respectively. From the plots, it can be seen that both linear and nonlinear multivariate regression based methods can estimate the slow part of the PM changes, but the details of the rapid changes cannot be estimated precisely. In contrast, the neural network-based method, with good nonlinear learning capability, captured the changes in PM concentration more completely and accurately.

**Figure 9:**
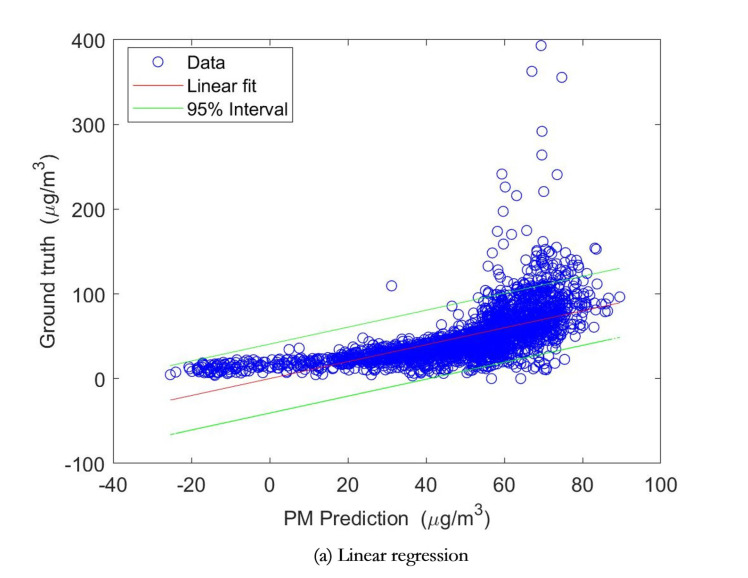

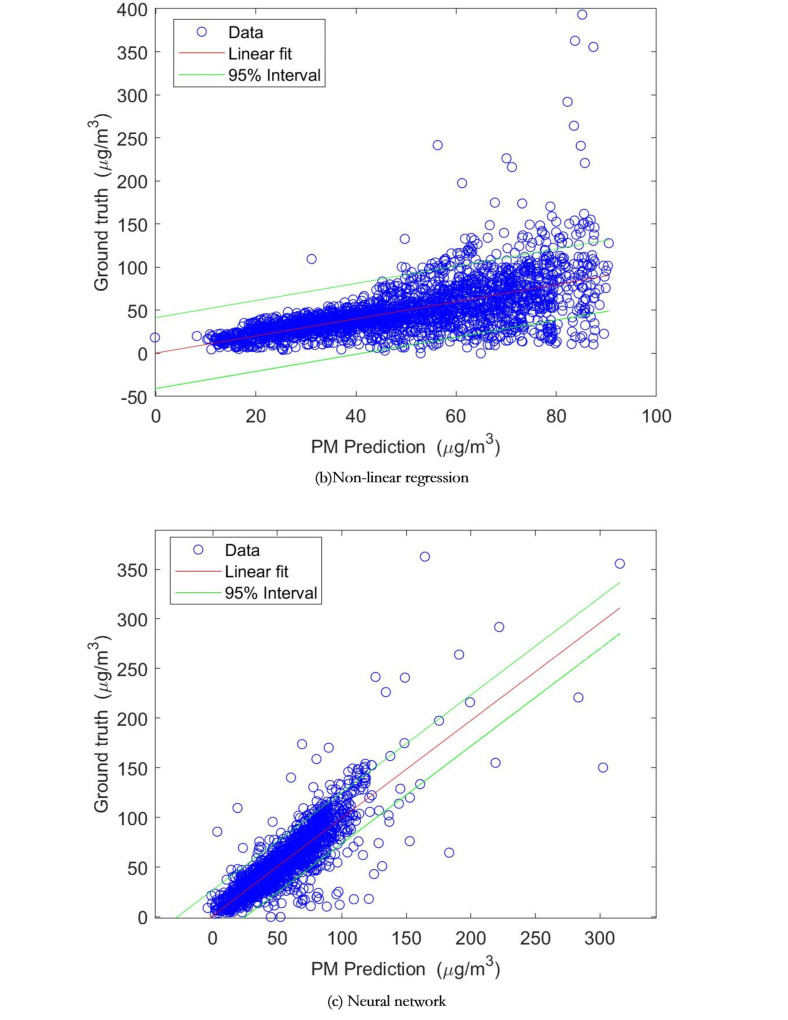
Linear regression lines with red color between estimated PM${}_{2.5}$ concentrations and the PM${}_{2.5}$ observation (reference) data. The spaces between the two green lines are the 95% prediction interval.

To further the performance of the three machine learning algorithms, the Pearson’s linear correlation was used for performing linear regressions between the output of models and ground truth data. The results were reported in Figure 9 and Table 2. The results showed that the multivariate linear regression method has the root mean square error (RMSE) of 24.6756 μg/m${}^{3}$ with a correlation coefficient of 0.6281, by referring to the ground truth of PM time series data; and the multivariate nonlinear regression method has the RMSE of 24.9191 μg/m${}^{3}$ with a correlation coefficient of 0.6184, while the neural network based method has the best performance, of which the RMSE of PM estimates is 15.6391 μg/m${}^{3}$ with a better correlation coefficient of 0.8701.

## 4. Discussion

This paper attempts to estimate the concentration of PM${}_{2.5}$ from meteorological parameters using three machine learning models to answer the question of whether it can be estimated and with what accuracy.

From Table 1, the correlation between PM${}_{2.5}$ concentration and visibility is –0.5639. The correlation coefficients between the estimated PM${}_{2.5}$ value and the PM${}_{2.5}$ reference value are 0.6281 and 0.6184, respectively, after multiple linear and nonlinear regression models (see Table 2). This indicates that the accuracy of PM${}_{2.5}$ estimation by these two models does not improve much. The main reason is that the nonlinear relationship between PM and meteorological parameters such as visibility, wind speed, wind direction, and humidity is complicated.

The estimation accuracy of the PM${}_{2.5}$ is greatly improved by the neural network model, with a correlation coefficient of 0.8701, which is better than our previous results by using the hidden Markov models [31]. The results demonstrate the ability of the neural network model to learn nonlinear relationships.

It is very interesting to note that, although the correlations between PM${}_{2.5}$ and atmospheric pressure, rainfall rate, and temperature are very low (see Table 1), the use of these meteorological parameters is critical to the performance of the neural network. Therefore, we further conducted extensive experiments to investigate the effects of using different meteorological parameter inputs on the RMSE and correlation coefficients of the three machine learning model estimates.

As shown in Table 3, the performance of the neural network approach increases significantly with increasing meteorological parameters. The performance of the linear regression model increases slightly with increasing meteorological parameters; however, the performance of the nonlinear regression model decreases considerably.

In machine learning, a very important issue is the problem of under-fitting and over-fitting of data. Unfortunately, the minimization of cost function for multivariate regression models suffers from poor generalization [40]. Therefore, the performance of the multivariate regression models for PM concentration prediction is limited. Our future work will introduce a regularization approach to further improve the performance of the multiple regression model. In contrast, Bayesian regularization has some promising advantages for the training of neural network models [40]. Bayesian regularization does not require a validation dataset; the method itself presents an evaluation of the evidence, which keeps the model from being overtrained. In addition, the Bayesian regularization method introduces Occam’s razor that automatically penalizes overly complex models, so the model is difficult to be overfitted [38,39]. One limitation of this study is that the PM and meteorological data are six-month time series. Our future work will collect data for longer periods to further evaluate the performance of the model, and consider other types of neural network models to further improve the accuracy of PM concentration predictions.

## 5. Conclusions

This paper demonstrates the potential of using multi-weather sensors to monitor PM${}_{2.5}$ concentrations. The accuracy of PM${}_{2.5}$ concentrations has been studied by using a comparison of three classical machine learning methods. The results show that the neural network-based approach outperforms both multivariate linear and nonlinear regression approaches, with encouraging results, a root mean square error of 15.6391 μg/m${}^{3}$ and a correlation coefficient of 0.8701. This study means that we can estimate the PM concentrations in real time from the high density network of automatic weather stations. Machine learning methods using data of these automated weather stations can provide new insights into mapping PM concentrations, and may make a valuable contribution to our understanding of the particles distribution and its cycle. Our future work will include acquiring more data and using other types of neural network models to further improve the accuracy of PM predictions.

## Figures and Tables

**Table 1:** Pearson’s linear correlation coefficient between PM${}_{2.5}$ and meteorological parameters.

Visibility	Wind Direction	Wind Speed	Relative Humidity	Temperature	Atmospheric Pressure	Rainfall Rate
−0.5639	0.2830	−0.2839	0.1201	−0.0424	0.0828	−0.0308

**Table 2:** PM${}_{2.5}$ prediction performances of three different models.

	Linear Regression	Nonlinear Regression	Neural Network
Averaged RMSE (μg/m${}^{3}$)	24.6756	24.9191	15.6391
Correlation coefficient	0.6281	0.6184	0.8701

**Table 3:** Effect of using different meteorological parameter inputs on the averaged RMSE (μg/m${}^{3}$) and correlation coefficients of three machine learning model outputs.

Parameters	Linear Regression	Nonlinear Regression	Neural Network
visibility + wind + RH	24.6756/0.6281	**24.9191/0.6184**	20.4548/0.7643
visibility + wind + RH + temperature	24.6670/0.6284	27.5901/0.5107	17.2791/ 0.8387
visibility + wind + RH + temperature + air pressure	24.5770/0.6319	30.1810/0.3093	17.1101/ 0.8460
visibility + wind + RH + temperature + air pressure + rainfall	**24.5141/0.6343**	29.0009/ 0.4132	**15.6391/ 0.8701**
